# Depressive symptoms in relation to overall survival in people with head and neck cancer: A longitudinal cohort study

**DOI:** 10.1002/pon.4816

**Published:** 2018-07-23

**Authors:** Femke Jansen, Irma M. Verdonck‐de Leeuw, Pim Cuijpers, C. René Leemans, Tim Waterboer, Michael Pawlita, Chris Penfold, Steven J. Thomas, Andrea Waylen, Andrew R. Ness

**Affiliations:** ^1^ Department of Otolaryngology‐Head and Neck Surgery, Cancer Center Amsterdam (CCA) VU University Medical Center Amsterdam The Netherlands; ^2^ Department of Clinical, Neuro and Development Psychology, Amsterdam Public Health Research Institute Vrije Universiteit Amsterdam Amsterdam The Netherlands; ^3^ Molecular Diagnostics of Oncogenic Infections Division German Cancer Research Center (DFKZ) Heidelberg Germany; ^4^ National Institute for Health Research (NIHR) Bristol Biomedical Research Centre the University Hospitals Bristol NHS Foundation Trust and the University of Bristol Bristol UK; ^5^ School of Oral and Dental Sciences University of Bristol Bristol UK

**Keywords:** cancer, depression, depressive symptoms, head and neck cancer, mortality, oncology, survival

## Abstract

**Objective:**

The objective of the study is to investigate the relation between pretreatment depressive symptoms (DS) and the course of DS during the first year after cancer diagnosis, and overall survival among people with head and neck cancer (HNC).

**Methods:**

Data from the Head and Neck 5000 prospective clinical cohort study were used. Depressive symptoms were measured using the Hospital Anxiety and Depression Scale (HADS) pretreatment, at 4 and 12‐month follow‐up. Also, socio‐demographic, clinical, lifestyle, and mortality data were collected. The association between before start of treatment DS (HADS‐depression > 7) and course (never DS, recovered from DS, or persistent/recurrent/late DS at 12‐month follow‐up) and survival was investigated using Cox regression. Unadjusted and adjusted analyses were performed.

**Results:**

In total, 384 of the 2144 persons (18%) reported pretreatment DS. Regarding DS course, 63% never had DS, 16% recovered, and 20% had persistent/recurrent/late DS. People with pretreatment DS had a higher risk of earlier death than people without DS (hazard ratio (HR) = 1.65; 95% confidence interval (CI) 1.33‐2.05), but this decreased after correcting for socio‐demographic, clinical, and lifestyle‐related factors (HR = 1.21; 95% CI 0.97‐1.52). Regarding the course of DS, people with persistent/recurrent/late DS had a higher risk of earlier death (HR = 2.04; 95% CI 1.36‐3.05), while people who recovered had a comparable risk (HR = 1.12; 95% CI 0.66‐1.90) as the reference group who never experienced DS. After correcting for socio‐demographic and clinical factors, people with persistent/recurrent/late DS still had a higher risk of earlier death (HR = 1.66; 95% CI 1.09‐2.53).

**Conclusions:**

Pretreatment DS and persistent/recurrent/late DS were associated with worse survival among people with HNC.

## 1 BACKGROUND

Clinical depression as well as depressive symptoms (DS) have been reported to increase mortality and reduce survival in different populations.^1^, ^2^, ^3^ Among people with different types of cancer, those with a clinical diagnosis of minor or major depression have a 39% higher risk of dying during the follow‐up period than people without depression.^1^ People with increased levels of DS, as measured using validated patient‐reported outcome measures, have a 25% higher risk of dying during the follow‐up period.^1^

People diagnosed with head and neck cancer (HNC) are prone to depression or DS.^4^, ^5^ Previous studies on the association between clinical depression^6^ or DS^7^, ^8^, ^9^, ^10^, ^11^, ^12^, ^13^ and survival in people with HNC reported mixed results. Some studies reported no association,^7^, ^8^ while others reported worse survival or higher mortality in people with depression or DS.^6^, ^9^, ^10^, ^11^, ^12^, ^13^ Half of these studies were, however, limited by small number of events (eg, disease‐related or overall deaths),^7^, ^9^, ^10^, ^11^, ^12^, ^13^ hampering the ability to account for different covariates in the survival analyses. In addition, most studies were limited to a single measurement of clinical depression or DS,^7^, ^8^, ^10^, ^11^, ^12^, ^13^ mostly prior to treatment.^7^, ^10^, ^11^, ^12^, ^13^ As previously reported,^14^ pretreatment DS may result from the short‐term response to cancer diagnosis and may not necessarily reflect a person's long‐term course of DS and, therefore, may be a less important associated factor of survival than DS at follow‐up.

A previous study reports that, in 40% of people with HNC, DS level indeed changed between the pretreatment and posttreatment measurement.^15^ Four different courses of DS were identified: people without DS, people who developed DS (33%), people who recovered from DS (7%), and people with persistent DS (4%). A recent study comparing survival outcomes of people with lung cancer reported on 4 comparable courses of DS.^16^ They found that people who developed or had persistent DS had an increased risk of earlier death, while people who recovered had the same risk as the reference group of people who never reported DS.

A recent large longitudinal study that measured depression more than once in people with HNC in relation to survival found that depression in the 2 years before HNC diagnosis as well as depression in the year after diagnosis was associated with worsened cancer‐specific and overall survival.^6^ In that study, however, no distinction was made between people who recovered from their depression during follow‐up and those who did not. In addition, depression was defined as a registered clinical depression diagnosis based on Medicare claims data. The generalizability of these findings to people with DS or undiagnosed depression is unclear.

This study, therefore, aimed to investigate the relation between pretreatment DS as well as the course of DS during the first year after cancer diagnosis and overall survival among people with HNC.

## 2 METHODS

### 2.1 Design and study population

In this study, data from the Head and Neck 5000 prospective clinical cohort study was used (dataset version 2.1),^17^, ^18^ including people with HNC from 76 centers in the United Kingdom. People with HNC were asked to participate if they had a new primary HNC diagnosis or were diagnosed with an unknown primary tumor likely to be HNC, and if they were ≥16 years. People were excluded if they did not have the capacity to provide informed consent or were too vulnerable for participation. In total, 5511 persons with HNC consented to participate from April 2011 to December 2014. For this particular study, we limited the population to people diagnosed with cancer of the oral cavity, oropharynx, hypopharynx, or larynx and those treated with curative intent. Besides, participants needed to have a baseline measurement of DS, and have complete socio‐demographic, clinical, and lifestyle‐related data.

All participants provided written informed consent. The study was approved by the National Research Ethics Committee (South West Frenchay Ethics Committee, reference 10/H0107/57, November 5, 2010), and approved by the research and development departments for participating NHS Trusts.

### 2.2 Measures

The English version of the Hospital Anxiety and Depression Scale (HADS) was used to assess psychological distress (HADS‐total), level of DS (HADS‐D), and level of anxiety symptoms (HADS‐A) before the start of treatment, and at 4 and 12‐month follow‐up.^19^, ^20^ A HADS‐D > 7 was used as a cutoff for identifying persons with DS.^21^ Internal consistency of the HADS‐D in this study was *α* = .851.

Study‐specific questions were used to measure pretreatment tobacco use and alcohol consumption. Tobacco use was categorized as current smoker, former smoker, or never smoked.^22^ For alcohol consumption, people were categorized as nondrinkers, moderate drinkers (1‐14 drinks per week), hazardous drinkers (14‐35 drinks/week for women and 14‐50 drinks/week for men), or harmful drinkers (>35 drinks/week for women and >50 drinks/week for men).^22^ In addition, age, gender, marital status, education level, annual household income, and deprivation status were measured. Deprivation status was measured using the Index of Multiple Deprivation (IMD) 2010.^23^

### 2.3 Clinical information

Clinical information was abstracted from the hospital information system and patients' notes by research nurses. Clinical information included the primary *International Classification of Diseases* (ICD) 2010 diagnosis, intended and actual received treatment, Adult Comorbidity Evaluation (ACE‐27), TNM‐stage, and human papilloma virus (HPV) status. Human papilloma virus status was based on serology data, and defined as positive where HPV16E6 was positive (>1000 median fluorescence intensity).^24^ At the start of the study, participants were flagged with the United Kingdom Health and Social Care Information Centre so that the study team was provided with information on overall mortality (mortality and mortality date).

### 2.4 Statistical analyses

All analyses were performed using the IBM Statistical Package for the Social Science (SPSS) version 23 (IBM Corp., Armonk, NY USA). Chi‐square tests and independent samples *t*‐test analyses were used to analyze differences between groups.

To assess the association between pretreatment DS and overall survival, a series of Cox regression analyses were performed. At first, minimally adjusted analyses adjusted for age and gender were performed. Analyses were performed in the total population as well as in people with oral cavity, HPV‐positive oropharyngeal, and HPV‐negative oropharyngeal and laryngeal cancer separately. Survival time was defined as days from date of consent to censoring or date of death. Besides these minimally adjusted analyses, we investigated whether potential associations remained after adjusting for socio‐demographic and clinical factors. Also, Cox regression analyses adjusted for lifestyle‐related factors were performed. Previous literature hypothesized that lifestyle may mediate the association between depression or DS and survival.^3^, ^25^ However, other studies added lifestyle as a potential confounder to the model.^7^, ^8^ Results can, therefore, be interpreted either as the direct effect after taking the potential mediating role of lifestyle into account or as the association that remains after adjusting for lifestyle as a potential confounder. Finally, post hoc analyses were performed by including each factor 1 by 1 to the minimally adjusted model, to investigate which factors had a strong influence on the association between DS and survival (defined as >10% change in hazard ratio (HR)). All categorical variables adhered to the proportional hazard assumption. Multicollinearity was not found.

Besides analyses on the association between pretreatment DS and survival, unadjusted and adjusted Cox regression analyses were performed using the course of DS in the first year after diagnosis as potential associated factor. For these analyses, people needed to have, besides the previously discussed eligibility criteria, complete HADS‐D at 4 and 12‐month follow‐up, and complete information on actual received treatment. All people were classified according to their course of DS^15^, ^16^: never DS (below threshold at all measurements), recovered from DS (above threshold at baseline and/or 4‐month follow‐up, but recovered at 12‐month follow‐up), or persistent/recurrent/late DS (above threshold at 12‐month follow‐up, regardless of outcome at baseline and 4‐month follow‐up). To prevent immortal time bias, landmark analyses with survival time defined as days between 12‐month follow‐up and date of censoring or death were performed.^26^, ^27^ Immortal time bias is bias resulting from misclassifying immortal time, ie, the time period during which the participants could not have been dead (in this case time between baseline and 12 months follow‐up), as survival time.

## 3 RESULTS

The HADS‐D score of the total study population (n = 2144) was on average 4.0 (standard deviation = 3.8, range 0‐21). Eighteen percent (n = 384) had pretreatment DS (Table 1, Appendices 1 and 2). Median follow‐up was 1046 days (range 601‐1963). Overall, 439 (20%) people died during the follow‐up period, of whom 332 were in the group without (19%) and 107 in the group with DS (28%). Mean survival time was 1509 days (95% confidence interval (CI) 1436‐1582) for the group with and 1651 days (95% CI 1620‐1682) for the group without DS.

**Table 1:** Characteristics of the groups with and without pretreatment depressive symptoms

Baseline Characteristics	Population Without Depressive Symptoms (HADS‐D ≤ 7)		Population with Depressive Symptoms (HADS‐D > 7)		
	n = 1760		n = 384		
	Frequency	Percentage	Frequency	Percentage	*P* Value
Socio‐demographic					
Age					.023
18**‐**50 years	229	13.0%	53	13.8%	
50‐64 years	868	49.3%	218	56.8%	
65‐79 years	583	33.1%	99	25.8%	
80 and older	80	4.5%	14	3.6%	
Gender					.267
Men	1353	76.9%	285	74.2%	
Women	407	23.1%	99	25.8%	
Marital status					.001
Single/widowed/divorced	550	31.3%	155	40.4%	
Married or living with a partner	1210	68.8%	229	59.6%	
Education level					.001
School education	777	44.1%	192	50.0%	
College	615	34.9%	143	37.2%	
Degree	368	20.9%	49	12.8%	
Annual household income					<.001
<£18 000	737	41.9%	233	60.7%	
£18 000‐£34 999	537	30.5%	101	26.3%	
>£35 000	486	27.6%	50	13.0%	
IMD quintiles					<.001
Low deprivation	762	43.3%	119	31.0%	
Moderate deprivation	401	22.8%	82	21.4%	
High deprivation	597	33.9%	183	47.7%	
Clinical					
Tumor location					.442
Oral cavity	503	28.6%	104	27.1%	
Oropharynx	800	45.5%	173	45.1%	
Hypopharynx	69	3.9%	22	5.7%	
Larynx	388	22.0%	85	22.1%	
Tumor stage					.028
Stage I	428	24.3%	66	17.2%	
Stage II	297	16.9%	69	18.0%	
Stage III	216	12.3%	53	13.8%	
Stage IV	819	46.5%	196	51.0%	
Intended treatment					.655
Surgery	558	31.7%	112	29.2%	
Radiotherapy	344	19.5%	73	19.0%	
Chemoradiation	595	33.8%	142	37.0%	
Surgery and adjuvant therapy	263	14.9%	57	14.8%	
Comorbidity					<.001
No comorbidity	883	50.2%	136	35.4%	
Mild decompensation	560	31.8%	132	34.4%	
Moderate/severe decompensation	317	18.0%	116	30.2%	
HPV status (oropharyngeal cancer only)^a^					.025
Positive	508	73.3%	95	64.2%	
Negative	185	26.7%	53	35.8%	
Lifestyle					
Tobacco usage					<.001
Current smoker	310	17.6%	112	29.2%	
Former smoker	1,012	57.5%	211	54.9%	
Never smoked	438	24.9%	61	15.9%	
Alcohol consumption					<.001
Nondrinker	412	23.4%	131	34.1%	
Moderate drinker	420	23.9%	51	13.3%	
Hazardous drinker	676	38.4%	133	34.6%	
Harmful drinker	252	14.3%	69	18.0%	

a HPV status is missing in 132 persons.

People with pretreatment DS had a higher risk of earlier death compared to people without DS (HR = 1.65; 95% CI 1.33‐2.05) (Table 2, Appendix 3). After adjustment for other socio‐demographic factors as well as for socio‐demographic and clinical factors, the strength of the association decreased (HR = 1.49; 95% CI 1.19‐1.86 and HR = 1.29; 95% CI 1.03‐1.62, respectively). After additional adjustment for potential mediation or confounding by lifestyle, the direct association further decreased (HR = 1.21; 95% CI 0.97‐1.52). Post hoc analyses showed that comorbidity (12% change), income (11% change), and smoking (10% change) had a major influence on the association.

**Table 2:** Cox regression analyses on the association between pretreatment depressive symptoms and overall survival

	All Head and Neck Cancers				Stratified															
	N = 2144				Oral Cavity Cancer N = 607				HPV+ Oropharyngeal Cancer N = 603				HPV− Oropharyngeal Cancer N = 238				Laryngeal Cancer N = 473			
	HR	95% CI		*P*	HR	95% CI		*P*	HR	95% CI		*P*	HR	95% CI		*P*	HR	95% CI		*P*
		Lower	Upper			Lower	Upper			Lower	Upper			Lower	Upper			Lower	Upper	
Base case analysis^a^																				
No depressive symptoms	Reference			<.001	Reference			.001	Reference			.479	Reference			.031	Reference			.021
Depressive symptoms	1.65	1.33	2.05		1.88	1.30	2.71		0.75	0.34	1.66		1.80	1.05	3.08		1.77	1.09	2.88	
Model adjusted for socio‐demographic characteristics^b^																				
No depressive symptoms	Reference			<.001	Reference			.013	Reference			.629	Reference			.108	Reference			.040
Depressive symptoms	1.49	1.19	1.86		1.62	1.11	2.36		0.82	0.37	1.82		1.59	0.90	2.79		1.70	1.02	2.81	
Model adjusted for socio‐demographic and clinical characteristics^c^																				
No depressive symptoms	Reference			.025	Reference			.052	Reference			.322	Reference			.306	Reference			.185
Depressive symptoms	1.29	1.03	1.62		1.46	1.00	2.14		0.66	0.29	1.50		1.35	0.76	2.40		1.43	0.84	2.41	
Model adjusted for socio‐demographic and clinical characteristics and for confounding/mediation by lifestyle^d^																				
No depressive symptoms	Reference			.094	Reference			.067	Reference			.304	Reference			.514	Reference			.247
Depressive symptoms	1.21	0.97	1.52		1.44	0.98	2.12		0.65	0.28	1.49		1.22	0.67	2.19		1.37	0.80	2.33	

HR, hazard ratio; 95% CI, 95% confidence interval; HADS‐D, Hospital Anxiety and Depression Scale‐Depression; HPV, human papilloma virus; HPV+, HPV‐positive; HPV−, HPV‐negative.

a The base case analysis is adjusted for age and gender.

b Adjusted for age, gender, marital status, education level, income, and IMD deprivation score.

c Adjusted for age, gender, marital status, education level, income, IMD deprivation score, tumor location, tumor stage, intended treatment, and comorbidity.

d Adjusted for age, gender, marital status, education level, income, IMD deprivation score, tumor location, tumor stage, intended treatment, and comorbidity, and for potential confounding/mediation by tobacco usage and alcohol consumption.

Subgroup analyses were performed for people with oral cavity, HPV‐positive oropharyngeal, and HPV‐negative oropharyngeal and laryngeal cancer. A higher risk of earlier death was found in people with oral cavity (HR = 1.88; 95% CI 1.30‐2.71) and HPV‐negative oropharyngeal (HR = 1.80; 95% CI 1.05‐3.08) and laryngeal cancer (HR = 1.77; 95% CI 1.09‐2.88) with DS, compared to people without DS, while no such association was found among people with HPV‐positive oropharyngeal cancer (HR = 0.75; 95% CI 0.34‐1.66) (Table 2). After additional adjustment, the strength of the associations decreased (Table 2).

### 3.1 Association between the course of depressive symptoms and overall survival

Of the 2144 people in the original sample, 1217 completed the HADS‐D at follow‐up (Appendix 1). The other 927 either died before the end of the first year (19%) or had missing follow‐up data (81%). Of the 1217 people, 445 (37%) experienced DS during the first year after treatment (13% pretreatment, 29% at 4‐month follow‐up, and 20% at 12‐month follow‐up). Regarding their course of DS in the first year after diagnosis: 63% were categorized as never had DS (n = 772), 16% as recovered from DS (n = 198), and 20% as having persistent/recurrent/late DS (respectively 7%, 1%, and 12%) (n = 247) (Appendix 1). The 3 groups differed on all characteristics, except gender (Appendix 4).

Median follow‐up from 12 months onwards was 676 days (range 236‐1598). In total, 123 (10%) people died during this follow‐up period, of whom 66 never had DS (9%), 18 had recovered from DS (9%), and 39 had persistent/recurrent/late DS (16%). Using people who never experienced DS as a reference group, it was found that those with persistent/recurrent/late DS had a HR of 2.04 (95% CI 1.36‐3.05), while people who recovered from DS had a comparable hazard as the reference group (HR = 1.12; 95% CI 0.66‐1.90) (Table 3 and Appendix 3). After adjustment for other socio‐demographic factors as well as for socio‐demographic and clinical factors, the HR of the group with persistent/recurrent/late DS compared to the reference group further decreased (HR = 1.88; 95% CI 1.25‐2.84 and HR = 1.66; 95% CI 1.09‐2.53). For the group who recovered from DS the findings remained stable (HR = 1.10, 95% CI 0.65‐1.86 and HR = 1.06; 95% CI 0.62‐1.83). Post hoc analyses showed that tumor location (18% change), comorbidity (17% change), and income (10% change) had a major influence on the association.

**Table 3:** Cox regression analyses on the association between the course of depressive symptoms and overall survival

Model	All Head and Neck Cancers N = 1217			
	HR	95% CI		*P* Value
		Lower	Upper	
Base case model (adjusted for age and gender)^a^				
Never depressive symptoms	Reference			.002
Recovered from depressive symptoms	1.12	0.66	1.90	
Persistent/recurrent/late depressive symptoms	2.04	1.36	3.05	
Model adjusted for socio‐demographic characteristics^b^				
Never depressive symptoms	Reference			.009
Recovered from depressive symptoms	1.10	0.65	1.86	
Persistent/recurrent/late depressive symptoms	1.88	1.25	2.84	
Model adjusted for socio‐demographic and clinical characteristics^c^				
Never depressive symptoms	Reference			.054
Recovered from depressive symptoms	1.06	0.62	1.83	
Persistent/recurrent/late depressive symptoms	1.66	1.09	2.53	

HR, hazard ratio; 95% CI, 95% confidence interval; HADS‐D, Hospital Anxiety and Depression Scale ‐Depression; HPV, human papilloma virus.

a The base case analysis is adjusted for age and gender.

b Adjusted for age, gender, marital status, education level, income, and IMD deprivation score.

c Adjusted for age, gender, marital status, education level, income, IMD deprivation score, tumor location, tumor stage, actual received treatment, and comorbidity.

## 4 DISCUSSION

Using data from the Head and Neck 5000 study,^17^, ^18^ it was found that 13% to 18% of people with HNC experience pretreatment DS. During the first year after diagnosis, 63% of people with HNC never had DS, 16% recovered from DS, and 20% had persistent/recurrent/late DS. Pretreatment DS and persistent/recurrent/late DS during the first year were found to be associated with worse overall survival among people with HNC.

This study showed that participants with pretreatment DS had a higher risk of earlier death compared to those without DS after adjusting for socio‐demographic and clinical factors. In addition, we found that, in people with oral cavity and HPV‐negative oropharyngeal and laryngeal cancer, DS were associated with worse survival, while in people with HPV‐positive oropharyngeal cancer, no such association was found. Previous studies on the association between pretreatment DS and overall survival have shown inconsistent results.^7^, ^10^, ^11^, ^12^, ^13^ Two studies in people with different types of HNC found no evidence for such an association (after adjustment),^7^, ^11^ while Shinn et al^10^ targeting people with oropharyngeal cancer, Zimmaro et al^12^ targeting people with mixed HNC treated with (chemo)radiation, and Chen et al also targeting people with mixed HNC reported an increased risk of earlier death or poorer overall survival among those with pretreatment DS. In contrast to our study, Shinn et al^10^ did not stratify for HPV status, as HPV status was only available for a subsample. Nevertheless, they reported no differences in HPV status between those with and without pretreatment DS, while we found such a difference. The inconsistent results of the different studies may be because of the limited statistical power resulting from small sample sizes (130 to 241 persons) in combination with low number of events (18 to 48 persons died during follow‐up).^7^, ^10^, ^11^, ^12^, ^13^ In our analyses, data from 2144 people were analyzed, of whom 439 (20%) died during the follow‐up period, which provided us with the opportunity to stratify our analyses and to adjust for a wide range of potential confounders. However, for the stratified analyses, additional analyses replicating our findings are warranted.

Besides worse survival in people with pretreatment DS, we also found that those with persistent/recurrent/late DS have higher risk of earlier death compared to the reference group of people who never experienced DS during the first year, while people who recovered from DS had the same risk. This is in line with results of a study among people with lung cancer.^16^ These findings suggest that, as previously hypothesized,^14^ people who have persistent DS or develop DS at follow‐up have worse survival.

The pathways via which DS may influence survival are still unclear.^1^, ^2^, ^3^, ^25^ A hypothesized pathway is that DS negatively influences lifestyle, which consequently worsens survival. To provide insight into the potential mediating role of lifestyle, we performed extra analyses in which we adjusted for tobacco and alcohol consumption. We found that after this adjustment, the strength of the association diminished, but remained evident. This suggests that lifestyle may explain part of the pathway between DS and survival, but not all. However, as lifestyle data were limited to pretreatment data, more research is needed on the causal role of lifestyle.

Another pathway may be that untreated depression can cause suicide.^25^ Although suicide is, compared to other diseases, relatively common among people with HNC,^28^ in absolute terms, it is a rare event. Also, tumor‐related and patient‐related biomarkers of endocrine, immune, and autonomic (dys)function or other clinical variables may explain the association between depression and survival.^25^ This might explain why we found a potential association between pretreatment DS and overall survival in people with HPV‐negative oropharyngeal cancer and not in HPV‐positive oropharyngeal cancer. However, future research is warranted to replicate these findings and to explore the specific role of HPV status and other biomarkers.

### 4.1 Study limitations

A limitation of this study was the missing data which may have influenced representativeness of findings and generalizability to the HNC population. Also, people with HNC were dichotomized based on a HADS‐D cutoff score of 7,^21^ while a score of 1 to 7 may already be indicative of mild DS. Finally, only information on DS and overall survival were available; further studies on clinical depression and disease‐specific survival are warranted.

### 4.2 Clinical implications

People with pretreatment DS as well as persistent/recurrent/late DS are at increased risk of earlier death. Previous studies have hypothesized that lifestyle and suicide may explain (part of) this association. Also, tumor‐related or patient‐related biomarkers are hypothesized to mediate this association.

## 5 CONCLUSION

Results of this study indicate that people with pretreatment DS as well as persistent/recurrent/late DS are at increased risk of earlier death. Further research is needed on potential pathways via which depression or DS may influence survival.

## CONFLICT OF INTEREST

None.

## APPENDIX 1 FLOW DIAGRAM

*927 HNC persons did not have complete HADS‐D data at 4 and 12‐month follow‐up, because they died before the end of the first year (19%) or dropped out or had missing data (81%).^1^ Never depressive symptoms (n = 772);^2^ Recovered from depressive symptoms before start of treatment or 4 months follow‐up (n = 198);^3^ Persistent/recurrent/late depressive symptoms at 12‐months follow‐up (n = 247).

## APPENDIX 2 COMPARISON OF PEOPLE WITH COMPLETE HADS‐D, SOCIO‐DEMOGRAPHIC, CLINICAL DATA, AND LIFESTYLE DATA (N = 2144), COMPARED TO PEOPLE WITH MISSING DATA (N = 2306)

**Table:**

Baseline Characteristics	Population with Complete Data		Population with Missing Data		
	n = 2144		n = 2306		
	Frequency	Percentage	Frequency	Percentage	*P* Value
Socio‐demographic					
Mean age (SD)	60.9 (10.5)		62.7 (11.0)		<.001
Gender					.062
Men	1,638	76.4%	1,706	74.0%	
Women	506	23.6%	600	26.0%	
Clinical					
Tumor location (ICD)					<.001
Oral cavity	607	28.3%	692	30.0%	
Oropharynx	973	45.4%	905	39.2%	
Hypopharynx	91	4.2%	125	5.4%	
Larynx	473	22.1%	584	25.3%	
Tumor stage					.622
Stage I	494	23.0%	509	22.1%	
Stage II	366	17.1%	419	18.2%	
Stage III	269	12.5%	303	13.2%	
Stage IV	1,015	47.3%	1,068	46.5%	
Missing	0		7		
Intended treatment					<.001
Surgery	670	31.3%	764	33.1%	
Radiotherapy	417	19.4%	507	22.0%	
Chemoradiation	737	34.4%	631	27.4%	
Surgery and adjuvant therapy	320	14.9%	404	17.5%	
Status					.008
Alive	1,705	79.5%	1,757	76.2%	
Died	439	20.5%	549	23.8%	

## APPENDIX 3 SURVIVAL CURVES

- Survival analysis on pretreatment depressive symptoms adjusted for socio‐demographic and clinical characteristics and potential mediation or confounding by lifestyle factors

- Survival analysis on the course of depressive symptoms adjusted for socio‐demographic and clinical characteristics

**Table:**

Pretreatment Depressive Symptoms					Course of Depressive Symptoms						
	Number at Risk (Number Censored) per Time Point					Number at Risk (Number Censored per Time Point)					
	0 days	500 days	1000 days	1500 days		0 days	250 days	500 days	750 days	1000 days	1250 days
No	1760 (0)	1584 (0)	811 (643)	170 (1261)	Never symptoms	772 (0)	752 (3)	528 (194)	308 (401)	143 (565)	47 (659)
Yes	384 (0)	322 (0)	160 (121)	35 (242)	Recovered from symptoms	198 (0)	191 (3)	120 (64)	70 (112)	38 (143)	12 (168)
					Persistent, recurrent or late symptoms	247 (0)	226 (1)	157 (58)	96 (116)	46 (165)	20 (189)

## APPENDIX 4 POPULATION CHARACTERISTICS OF THE GROUPS WITH DIFFERENT COURSES OF DEPRESSIVE SYMPTOMS

**Table:**

	Never Depressive Symptoms^a^ N = 772		Recovered from Depressive Symptoms^b^ N = 198		Persistent/Recurrent/Late Depressive Symptoms 12‐month Follow‐Up^c^ N = 247		
	Frequency	Percentage	Frequency	Percentage	Frequency	Percentage	*P* Value
Socio‐demographic							
Age							<.001
18‐50 years	80	10.4%	19	9.6%	27	10.9%	
50‐64 years	360	46.6%	120	60.6%	148	59.9%	
65‐79 years	292	37.8%	51	25.8%	67	27.1%	
80 and older	40	5.2%	8	4.0%	5	2.0%	
Gender							.564
Men	591	76.6%	145	73.2%	184	74.5%	
Women	181	23.4%	53	26.8%	63	25.5%	
Marital status							.016
Single, widowed or divorced	205	26.6%	51	25.8%	88	35.6%	
Married/living with a partner	567	73.4%	147	74.2%	159	64.4%	
Highest education level							.001
School education	302	39.1%	83	41.9%	128	51.8%	
College	289	37.4%	62	31.3%	85	34.4%	
Degree	181	23.4%	53	26.8%	34	13.8%	
Annual household income							<.001
Less than £18 000	280	36.3%	69	34.8%	137	55.5%	
£18 000‐£34 999	244	31.6%	69	34.8%	74	30.0%	
More than £35 000	248	32.1%	60	30.3%	36	14.6%	
IMD quintiles (2010)							<.001
Low deprivation	387	50.1%	92	46.5%	86	34.8%	
Moderate deprivation	171	22.2%	45	22.7%	54	21.9%	
High deprivation	214	27.7%	61	30.8%	107	43.3%	
Clinical							
Tumor location (ICD)							<.001
Oral cavity	252	32.6%	34	17.2%	63	25.5%	
Oropharynx	302	39.1%	138	69.7%	127	51.4%	
Hypopharynx	18	2.3%	5	2.5%	9	3.6%	
Larynx	200	25.9%	21	10.6%	48	19.4%	
Tumor stage							<.001
Stage I	249	32.3%	18	9.1%	47	19.0%	
Stage II	143	18.5%	22	11.1%	49	19.8%	
Stage III	94	12.2%	24	12.1%	33	13.4%	
Stage IV	286	37.0%	134	67.7%	118	47.8%	
Actual received treatment							<.001
Surgery	221	28.6%	20	10.1%	51	20.6%	
Radiotherapy	162	21.0%	22	11.1%	43	17.4%	
Chemoradiation	221	28.6%	118	59.6%	100	40.5%	
Surgery and adjuvant therapy	168	21.8%	38	19.2%	53	21.5%	
Comorbidity index							<.001
No comorbidity	407	52.7%	106	53.5%	94	38.1%	
Mild decompensation	250	32.4%	62	31.3%	84	34.0%	
Moderate/severe decompensation	115	14.9%	30	15.2%	69	27.9%	
Lifestyle							
Tobacco usage							<.001
Current smoker	94	12.2%	25	12.6%	62	25.1%	
Former smoker	467	60.5%	110	55.6%	137	55.5%	
Never smoked	211	27.3%	63	31.8%	48	19.4%	
Alcohol consumption							.001
Nondrinker	168	21.8%	45	22.7%	76	30.8%	
Moderate drinker	207	26.8%	46	23.2%	37	15.0%	
Hazardous drinker	301	39.0%	72	36.4%	92	37.2%	
Harmful drinker	96	12.4%	35	17.7%	42	17.0%	

a HADS‐D below threshold at all measurements.

b HADS‐D above threshold at baseline and/or 4‐month follow‐up, but recovered at 12‐month follow‐up.

c HADS‐D above threshold at 12‐month follow‐up, regardless of outcome at baseline and 4‐month follow‐up.
